# Oligoprogression of Solid Tumors on Immune Checkpoint Inhibitors: The Impact of Local Ablative Radiation Therapy

**DOI:** 10.3390/biomedicines10102481

**Published:** 2022-10-05

**Authors:** Kunal K. Sindhu, Anthony D. Nehlsen, Eric J. Lehrer, Jared P. Rowley, Richard G. Stock, Matthew D. Galsky, Michael Buckstein

**Affiliations:** 1 Department of Radiation Oncology, Icahn School of Medicine at Mount Sinai, New York, NY 10029, USA; 2 Department of Radiation Oncology, Maimonides Medical Center, Brooklyn, NY 11220, USA; 3 Tisch Cancer Institute, Icahn School of Medicine at Mount Sinai, New York, NY 10029, USA; 4 Division of Hematology and Medical Oncology, Department of Medicine, Icahn School of Medicine at Mount Sinai, New York, NY 10029, USA

**Keywords:** immunotherapy, oligoprogression, radiation therapy

## Abstract

The breakthrough of a limited number of clones while on immune checkpoint inhibitors (ICIs), known as oligoprogression, has been previously described. The benefit of ablative radiation therapy (RT) directed at these clones, as opposed to changing systemic therapy, is unclear. We analyzed 30 patients with advanced solid tumors, the majority of whom (23/30, 86.7%) had either hepatocellular or urothelial carcinoma, who experienced oligoprogression on ICIs and were referred for RT. In this study, oligoprogression was defined as having experienced progression at three or fewer metastatic sites outside of the brain after achieving at least stable disease on ICIs for a minimum of three months. The median time to oligoprogression was 11.1 months from the initiation of immunotherapy. 24 patients had one oligoprogressive lesion and six had two. The median radiation dose delivered was 4650 cGy in a median of five fractions. The median progression-free survival (PFS) after RT was 7.1 months, and the time to oligoprogression was not a significant predictor of PFS2. 26 patients continued on ICIs after RT. While 17 patients subsequently progressed, 15 did so at three or fewer metastatic sites and could have theoretically stood to benefit from an additional course of salvage RT to further extend the lifespan of their ICIs. Overall survival at 6, 12, and 24 months was 100.0%, 96.3%, and 82.8%, respectively. These results suggest that RT may provide a PFS benefit and extend the lifespan of ICIs in patients who experience oligoprogression. Regardless of PFS, however, overall survival in this population appears to be excellent.

## 1. Introduction

Immune checkpoint inhibitors (ICIs) have been shown to have activity in patients with a wide variety of malignancies and have dramatically transformed the field of oncology in recent years [1]. Since ICIs have a very different mechanism of action from cytotoxic chemotherapy and targeted therapies, different patterns of recurrence and progression have been characterized [2,3]. Research is ongoing to determine whether these differences may be exploited through the development of novel treatment protocols [2].

While a significant fraction of patients on ICIs experience durable disease control, many ultimately experience disease progression [4,5]. A certain subset of these patients experience oligoprogression, a condition marked by the progression of metastatic disease at a limited number of anatomic sites [6]. The term has yet to be universally defined and its overall incidence in the population of patients on ICIs, which varies depending on the precise definition of oligoprogression that is employed, is unclear. However, a prior analysis examining 425 patients on ICIs at a large institution found that 4.1% of them experienced progression at three or fewer metastatic sites after achieving at least stable disease for a minimum of three months on ICIs [7]. In addition, its incidence was substantially higher in patients who achieved only stable disease as a best response to ICIs.

The optimal management of patients with oligoprogression is unclear. Oftentimes, the oncological care team must weigh whether to change such a patient’s systemic therapy or selectively treat the refractory clones (with local therapy) while maintaining the current ICI. Prior work has suggested that local therapy may provide a benefit to patients with oligoprogressive disease on other systemic therapies. Several studies, for example, have noted a possible progression-free survival (PFS) benefit associated with local therapy in patients with non-small cell lung cancer (NSCLC) who experience oligoprogression on tyrosine-kinase inhibitors [8,9,10,11,12]. Secondly, an interim analysis of the CURB trial, in which patients with metastatic breast cancer and NSCLC who progressed at five or fewer disease sites on systemic therapy and were randomized to receive stereotactic body radiation therapy (SBRT) and palliative care or palliative care alone, found a PFS benefit in the SBRT arm. However, the authors noted that this benefit was entirely driven by patients with NSCLC; among patients with breast cancer, there was no difference in PFS between the two treatment arms [13]. Additionally, local therapy can potentially benefit patients with oligometastatic disease, as well. A recent systematic review by Zamagni et al. evaluating the potential benefit of SBRT in patients with nodal oligometastatic disease secondary to prostate cancer, for example, found that SBRT was safe, offered high rates of local control, and allowed for a delay in the initiation of androgen deprivation therapy [14]. Given the potential mechanistic differences in oligoprogression in patients on ICIs, local therapy may be of particular benefit to this patient population. Indeed, early work examining patients with NSCLC receiving ICIs found that radiation therapy offers high rates of local control at oligoprogressive disease sites and may prolong their response to ICIs [15].

These results suggest that oligoprogressive disease in patients on ICIs may represent a distinct form of disease progression requiring its own specific management strategies centered upon the use of local therapies. The precise benefits of local therapies in these patients, however, is still being defined. In this study, we analyzed a group of patients with advanced solid tumors who experienced oligoprogression on ICIs and were treated with ablative radiotherapy. We then sought to evaluate the efficacy of RT in potentially prolonging PFS and overall survival (OS) in this population of patients.

## 2. Materials and Methods

The Program for Protection of Human Subjects at the Icahn School of Medicine at Mount Sinai approved this study. We identified 30 patients with advanced solid tumors, excluding glioblastoma multiforme, on ICIs (atezolizumab, ipilimumab, nivolumab, or pembrolizumab) who experienced oligoprogression and were referred for salvage radiation therapy at our institution between 2011 and 2020. Oligoprogression was defined as progression at three or fewer sites of metastatic disease outside of the brain after achieving at least stable disease on ICIs for a minimum of three months on cross-sectional imaging. Best responses to ICIs were recorded as either a complete response (CR), partial response (PR), or stable disease (SD) as per the treating physician. Discrete lesions within the same organ or lymph node chain were counted individually as separate lesions.

We then calculated the time to oligoprogression, defined as the time from the day of initiation of an ICI to the day of oligoprogression, and estimated the time to second PFS (PFS2) among these patients via a Kaplan–Meier survival analysis. For the purposes of this study, PFS2 was defined as the time from the last day of radiation therapy to the day of second progression, death, or latest follow-up. OS was also recorded, as defined from the last day of radiation therapy to the time of death or last follow-up.

RStudio (Version 1.1.383, Boston, MA, USA) was used to conduct the statistical analyses. The ‘survminer’ package was used to conduct the Kaplan–Meier analyses for overall survival and PFS2. The log-rank test was used to compare PFS2 among patients with CRs and PRs, where the null hypothesis was rejected for *p* < 0.05. A univariate logistic regression model was used to assess the impact of the time to oligoprogression on PFS2. Graphpad Prism (Version 9.3.1, La Jolla, CA, USA) was used to generate the swimmer’s plot.

## 3. Results

Baseline characteristics of the patients we identified are seen in Table 1. The best response on ICIs of four patients (13.3%) was a CR, sixteen patients (53.3%) a PR, and ten patients (33.3%) SD. The median time from the initiation of immunotherapy to oligoprogression (and hence before irradiation) among this group of patients was 11.1 months (range 3.5–32.8 months).

There were 24 patients (80.0%) in this series who had one oligoprogressive lesion and six (20.0%) who had two oligoprogressive lesions (Table 2). The most common oligoprogressive sites were lymph nodes (13 lesions, 10 patients), the liver (9 lesions, 8 patients), and the adrenal glands, bones, and lungs (2 lesions, 2 patients each). A total of 16 patients (53.3%) experienced oligoprogression at a site of disease that did not exist prior to initiating an ICI (new site), while 14 (46.7%) experienced oligoprogression at a site of disease that did exist prior to initiating an ICI (old site). All patients received radiation therapy to a median dose of 4650 cGy (range 1800–6000 cGy) in a median of five fractions (range 1–30 fractions). A total of 26 patients (86.7%) continued on the same ICI after completing radiation therapy; in four cases, the treating oncologist, in consultation with the patient, chose to transition to another systemic therapy after RT.

**Figure 1:**
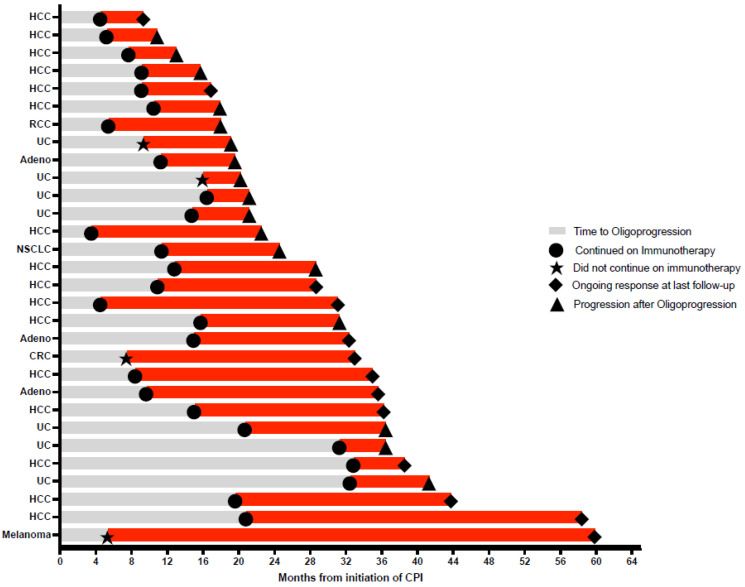
Disease courses in patients who achieved at least stable disease on ICIs for at least three months and then experienced oligoprogression. Adeno, adenocarcinoma; CRC, colorectal carcinoma; HCC, hepatocellular carcinoma; NSCLC, non-small cell lung cancer; RCC, renal cell carcinoma; UC, urothelial carcinoma.

**Figure 2:**
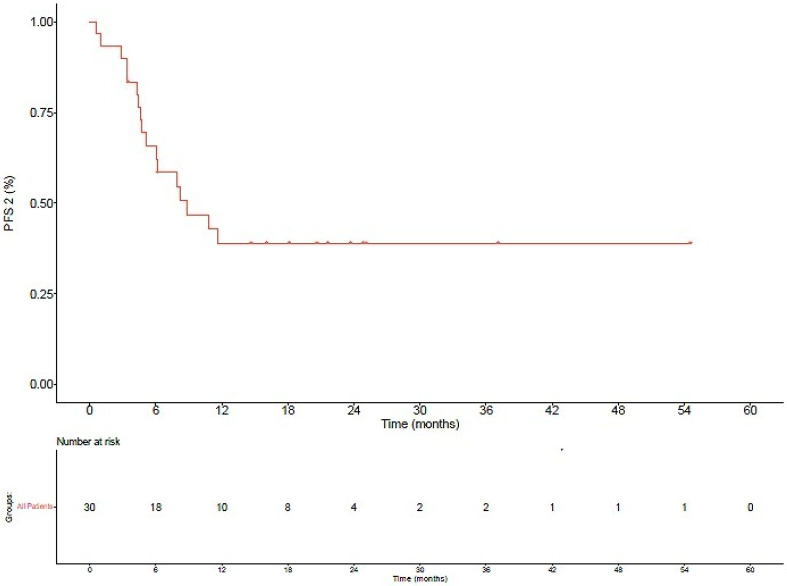
PFS2 among all oligoprogressors who received radiation therapy.

**Figure 3:**
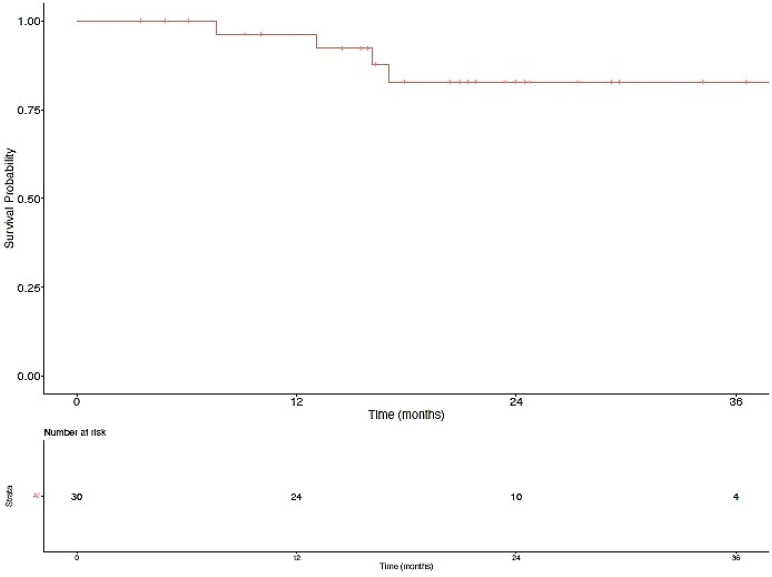
OS among all oligoprogressors who received radiation therapy.

The median follow-up for the patients in this series was 36.1 months (range 9.5–77.0 months) from the initiation of immunotherapy. The median PFS2 was 7.1 months (Range 0.6–54.6 months, Figure 1 and Figure 2). There was no statistical difference in the median PFS2 between patients who experienced oligoprogression at a new versus an old site of disease (presumably representing progression of an existing metastatic site) (*p* = 0.73). Additionally, there was no difference in the median PFS2 between patients whose best response on an ICI was a CR or PR versus those who experienced SD (*p* = 0.13). Time to oligoprogression was not a significant predictor of PFS2 (odds ratio 1.02, 95% confidence interval 0–1.13, *p* = 0.68). Thirteen of the thirty patients (43.3%) had not subsequently progressed, and four of the thirty patients (13.3%) had died at the time of this analysis. Overall survival at 6, 12, and 24 months was 100.0%, 96.3%, and 82.8%, respectively (Figure 3). Radiation therapy was tolerated quite well overall, with only three patients (10%) experiencing grade II and zero patients experiencing grade III or higher acute toxicities.

**Figure 4:**
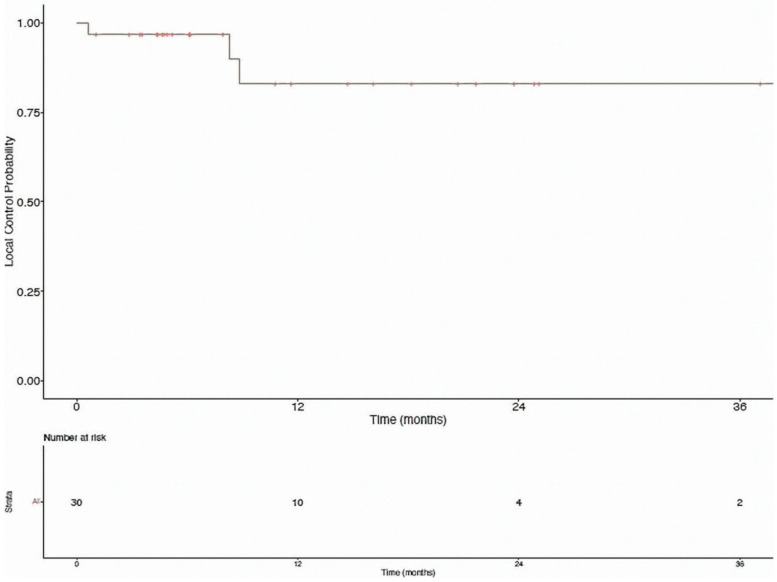
Local control of oligoprogressive sites treated with radiation therapy.

Seventeen of the thirty patients (56.7%) who received radiation therapy subsequently progressed, with a median time to second progression of 8.6 months (range 4.6–19.4 months) after completing treatment. Five of these patients progressed within six months of completing radiation therapy. Six patients progressed at a single site, four at two sites, five at three sites, and two at four or more sites. The most common sites of second progression included the liver (14 sites, 7 patients) and lymph nodes (13 sites, 8 patients). 14/17 patients (82.4%) who progressed after receiving radiation therapy did so at a separate site, eight of whom subsequently received radiation therapy to the new site of disease progression; only three of the seventeen patients (17.6%) progressed at a treated site. Local control at treated, oligoprogressive sites at 6, 12, and 24 months was 96.7%, 82.9%, and 82.9%, respectively (Figure 4). More information on each patient in this cohort may be found in the supplementary Table S1.

Hematological parameters, including the absolute neutrophil count (ANC) and the neutrophil-to-lymphocyte ratio (NLR), were available for 29/30 patients. The mean ANC before and after RT was 3760 cells/microliter and 3371 cells/microliter, respectively, and the mean NLR before and after RT was 3.20 and 4.79, respectively. There was no significant correlation between the change in ANC or NLR before and after RT and the PFS2 (r = 0.36, *p* = 0.06 and r = 0.04, *p* = 0.84, respectively). Additionally, there was no significant difference in the mean ANC and NLR prior to RT among patients whose best response to treatment with an ICI was a CR, PR, or SD (*p* = 0.15 and *p* = 0.72, respectively).

## 4. Discussion

Historically, a central tenet of managing patients with advanced cancers is to modify systemic therapy upon progression of disease. This principle rests on the assumption that the underlying malignant cells in such patients have acquired mutations that have made them permanently resistant to existing systemic therapy [16]. Under this framework, continuing therapy in patients who have progressed could allow malignant cells that have developed drug-resistance mutations to proliferate, putting patients at-risk of poor outcomes [17].

This theory rests on several observations. In vitro studies in the 1970s showed that exposing tumor cells to high concentrations of chemotherapeutic agents could generate multi-drug resistant clones [18,19,20]. More recently, studies of the genomes of malignant cells have identified numerous mutations that encode drug-acquired resistance [21,22]. It is no surprise, then, that clinical trial design traditionally dictates that systemic therapy should be modified upon progression of disease.

However, in recent years, there has been a growing appreciation that not all patients with metastatic cancer have a similar clinical course. In fact, in some patients, an oligometastatic state might exist at diagnosis, in which local ablative therapy to a small number of metastases might produce superior outcomes compared to systemic therapy alone [23,24]. Other patients, by contrast, who experience at least stable disease on systemic therapy may develop a small number of sites that are progressive. The precise mechanisms by which this occurred in our cohort of patients was not explored. However, prior work has shown that progression events in patients on ICIs may be driven by changes in or loss of expression of the target antigen(s); the growth of a previously quiescent, de novo resistant subclone; or an evolution in the function of the immune system itself [25]. Even as these sites progress, other disease sites may remain controlled by the ICI. Thus, in these patients with oligoprogression, emerging data suggest that there may exist an opportunity to deliver local therapy to these sites as a way to extend the lifespan of the systemic therapy in question.

Local therapy may be of particular benefit to patients on ICIs. In these patients, local therapy may eliminate clones that evade antitumor immunity at oligoprogressive sites, while immune cells with the capacity for memory continue to limit the growth of other sites of disease. Prior studies have described an extended PFS in patients with melanoma and NSCLC on ICIs who receive local therapy [26,27]. Radiation therapy, in particular, is an attractive option for these patients. It is, by nature, non-invasive and, unlike other forms of local therapy, available as a treatment option to most patients, including those with poor performance statuses or significant comorbidities. It is also generally well-tolerated, as evidenced by the fact that zero patients in this series experienced grade III or higher acute toxicity.

We have previously examined outcomes in a cohort of 16 patients on ICIs who experienced oligoprogression and received a variety of different local treatments (including cryoablation, RT, surgery, transarterial chemoembolization, and radioembolization with yttrium-90) to such sites. In this study, in contrast, we describe outcomes in a larger, heterogeneous cohort of patients, with a longer period of follow-up, treated with ICIs and who only received radiation therapy to oligoprogressive sites of disease. We found that the median time to oligoprogression among these patients was 11.1 months from the initiation of immunotherapy. The vast majority of patients had only one site of oligoprogressive disease, and the median PFS2 was 7.1 months after completing RT. Crucially, the time to oligoprogression was not a significant predictor of PFS2, suggesting that RT could potentially even benefit patients who experience oligoprogression shortly after beginning ICIs. Additionally, most patients (26/30) in our cohort were able to continue on the same immunotherapeutic agent after completing RT. Interestingly, of the 17 patients who subsequently progressed after receiving RT, 15 experienced progression at three or fewer sites of disease. These patients could have theoretically stood to benefit from an additional course of salvage RT to further extend the lifespan of their ICIs. Regardless, the OS of this population is excellent, suggesting that oligoprogressive patients do very well. However, based on this small, retrospective cohort with referral biases, it is impossible to definitively assess how much radiation therapy helps these patients.

The strengths of this study include its relatively large sample size for studies of this nature and a relatively long follow-up period (>3 years). In contrast, there are several limitations of this analysis. While multiple histologies are included, the majority of patients (23/30, 86.7%) had either hepatocellular or urothelial carcinoma. While this likely reflects referral patterns at our institution, it might limit the study’s generalizability. In the case of hepatocellular carcinoma, in particular, multiple local therapy options, including radiofrequency ablation, RT, and transarterial therapies, exist. While there has historically been sparse literature examining outcomes in patients who receive radiation therapy for oligometastatic and oligoprogressive hepatocellular and urothelial carcinoma, that is slowly changing [28,29]. Secondly, while we defined oligoprogression as progression at three or fewer sites of metastatic disease outside of the brain after achieving at least stable disease on ICIs for a minimum of three months on cross-sectional imaging, other studies have utilized differing definitions, including by varying the number of progressive sites needed to qualify as oligoprogression and including progressive lesions in the brain. At this juncture, it is unclear which definition of oligoprogression is most clinically significant, and more research will be needed to provide oncologists with better guidance on when to consider disease progression as either oligoprogression (and thus consider local therapy) or widespread progression (and thus switch systemic therapy). Thirdly, the dose and fraction size of the radiation treatments delivered in this study varied. Fourth, the patients in this study were on multiple ICIs. It is as yet unclear if these ICIs necessarily offer patients equivalent long-term outcomes, and we cannot assume that all patients who receive RT for oligoprogression on various ICIs will experience equivalent treatment outcomes.

Moreover, few definitive conclusions may be drawn from a single institutional, retrospective study, and further research is warranted to ascertain the impact of radiation therapy on this specific patient population. While overall survival among patients in this study was high, the precise impact of radiation therapy on this metric is unclear and it is possible that these patients would have experienced favorable outcomes on systemic therapy alone. It is unclear, in fact, to whom patients who received RT in this study should be compared to in order to evaluate the efficacy of RT. Given the relative infrequency of oligoprogressive disease in patients on ICIs, obtaining data from prospective trials with sufficient numbers of enrolled patients is a challenge [7]. However, previous work may provide some hints as to the potential benefit of RT. An analysis of KEYNOTE-047, which randomized patients with metastatic NSCLC to either pembrolizumab + chemotherapy or placebo + chemotherapy found a median time to first progression of 8.0 months and median time to second progression of just 13.8 months from the initiation of treatment in the pembrolizumab group [30]. Another May 2021 multicenter analysis of patients with NSCLC and PD-L1 expression ≥50% who progressed on first line pembrolizumab monotherapy found that those patients who received local ablative treatments lived longer after progressing than those who switched systemic therapy or continued on pembrolizumab alone (overall survival of 13.9 months versus 8.0 months versus 8.2 months, respectively) [31]. Lastly, in the aforementioned CURB trial, the median PFS was just 10 weeks in the palliative standard of care arm versus 22 weeks in the SBRT arm; however, it should be noted that systemic therapy was given per the treating physician’s discretion and immunotherapy was not required for enrollment [13]. Thus, the lack of concrete data with which to compare our outcomes points to the need for further research on this topic.

## 5. Conclusions

Oligoprogression may represent a distinct disease state in patients on ICIs. Our results suggest that the use of radiation therapy to oligoprogressive sites of disease may provide a PFS benefit and extend the lifespan of ICIs. However, further study is necessary to optimize treatment strategies in this population of patients.

## Figures and Tables

**Table 1:** Baseline characteristics of patients who experienced oligoprogression by best response to immune checkpoint inhibition.

	Patients Experiencing Oligoprogression (n = 30)	
	CR/PR	SD
Number of patients	20	10
Sex, No. (%)		
Male	14 (70%)	5 (50%)
Female	6 (30%)	5 (50%)
Age at diagnosis in years, mean (STD)	63.1 (8.6)	59.2 (13.1)
Age at ICI initiation in years, mean (STD)	65.2 (6.8)	63.7 (12.8)
Race, No. (%)		
White	7 (35%)	4 (40%)
Hispanic	5 (25%)	2 (20%)
Black	5 (25%)	1 (10%)
Asian	3 (15%)	3 (30%)
ICI, No. (%)		
Nivolumab	14 (70%)	6 (60%)
Pembrolizumab	5 (25%)	3 (27%)
Atezolizumab	1 (5%)	1 (5%)
Ipilimumab	1 (5%)	0 (0%)
Other	0 (0%)	0 (0%)
Histology, No. (%)		
Hepatocellular carcinoma	13 (65%)	3 (27%)
Urothelial carcinoma	3 (15%)	4 (27%)
Adenocarcinoma	3 (15%)	0 (0%)
Colorectal Cancer	1 (5%)	0 (18%)
Melanoma	0 (0%)	1 (10%)
Non-small cell lung cancer	0 (0%)	1 (10%)
Renal cell carcinoma	0 (0%)	1 (10%)

CR, complete response; PR, partial response; SD, stable disease; STD, standard deviation; ICI, immune checkpoint inhibitor. Please note that one patient received dual immune checkpoint blockade with ipilimumab + nivolumab and thus the number of ICIs = 31 instead of 30.

**Table 2:** List of oligoprogressive cases.

Histology	Number of Oligoprogressive Lesions	Site(s) of Oligoprogression	Did Oligoprogression Occur in New Disease Sites or Sites That Existed Prior to ICI Initiation?	Radiation Dose	Number of Fractions	Did Patient Progress after Local Treatment?
HCC	1	Liver	New	4000	5	No
HCC	1	Porta hepatis LN	Old	4500	5	Yes
HCC	1	Porta hepatis LN	New	4500	5	Yes
HCC	2	Left crus, Left adrenal gland	Old	5000	5	Yes
HCC	1	Liver	Old	5000	5	No
HCC	2	Aortocaval LNs × 2	Old	6000	15	Yes
RCC	1	Right kidney	Old	4800	3	Yes
Urothelial Carcinoma	1	Left inguinal LN	New	3000	5	Yes
Adenocarcinoma	1	Para-aortic LNs	New	5500	25	Yes
Urothelial Carcinoma	1	Celiac LN	New	5000	10	Yes
Urothelial Carcinoma	1	Anterior abdominal wall	New	4500	5	Yes
Urothelial Carcinoma	2	Bladder and kidney	Old	6000	30	Yes
HCC	2	Liver	New	4000	5	Yes
NSCLC	1	L3	New	1800	1	Yes
HCC	1	Extrahepatic mass	Old	5000	20	Yes
HCC	1	Liver	New	5000	5	No
HCC	1	Portocaval LN	Old	4500	5	No
HCC	1	Liver	Old	4500	5	Yes
Adenocarcinoma	1	Right adrenal gland	Old	4800	4	No
Colorectal Cancer	1	Left Lung	New	4800	3	No
HCC	1	L3	New	2400	3	No
Adenocarcinoma	1	Liver	New	5000	5	No
HCC	1	Liver	New	5000	5	No
Urothelial Carcinoma	2	Left inguinal LN, Left obturator LN	New	2500	5	Yes
Urothelial Carcinoma	2	Left inguinal LN, Left obturator LN	New	2500	5	Yes
HCC	1	Liver	Old	4500	15	No
Urothelial Carcinoma	1	Left nephrectomy bed	Old	5000	5	Yes
HCC	1	Right lung	New	5000	5	No
HCC	1	Liver	Old	4500	5	No
Melanoma	1	Right intraparotid LN	Old	3000	5	No

RT, radiation therapy; LN, lymph node.

## Data Availability

Data are contained within the article.

## Supplementary Materials

The following supporting information can be downloaded at: https://www.mdpi.com/article/10.3390/biomedicines10102481/s1, Table S1: Charactersitics of and treatments received by the patients who experienced oligoprogression in this study.
