# Supplementary material for: Oligoprogression of Solid Tumors on Immune Checkpoint Inhibitors: The Impact of Local Ablative Radiation Therapy
Source: Biomedicines. 2022 Oct 5;10(10):2481. doi: 10.3390/biomedicines10102481 (PMC9599608; doi:10.3390/biomedicines10102481)
Supplement: Supplementary file 1 [file biomedicines-10-02481-s001.zip › biomedicines-1907062-supplementary.pdf]

Supplementary Table S1. Charactersitics of and treatments received by the patients who experienced oligoprogression in this study.

Patient	Diagnosis	Sex	Age at Diagnosis	Disease stage at ICI start	Number of disease sites pre-ICI	Previous Therapy	ICI Administered	Best Response on ICI	Time to Oligorogression on ICI (months)	Number of oligoprogressive lesions	Site(s) of oligoprogression	Did oligoprogression occur in site that existed prior to ICI initiation?	Radiation Dose	Number of Fractions	Progression after local treatment?	Salvage Treatments
1	HCC	Male	46.6	Recurrent	3	Surgery, TACE/RFA	Nivolumab	PR	10.5	2	Aortocaval LNs x 2	Old	6000	15	Yes	None
2	HCC	Male	69.2	Recurrent	3	Y90	Nivolumab	PR	5.2	1	Porta hepatis LN	Old	4500	5	Yes	Lenvantinib
3	Adenocarcinoma	Female	50.6	Recurrent	9	Surgery + FOLFOX, FOLFIRI + bevacizumab	Nivolumab	PR	9.6	1	Liver	New	5000	5	No	None
4	HCC	Male	68.7	Recurrent	3	TACE	Nivolumab	PR	4.5	1	Portocaval LN	Old	4500	5	No	None
5	Urothelial Carcinoma	Female	72.3	III	0	Surgery	Nivolumab	SD	14.7	1	Anterior abdominal wall	New	4500	5	Yes	Erdafitinib, enfortumab vedotin
6	HCC	Male	72.5	IV	5	None	Nivolumab	PR	8.4	1	L3	New	2400	3	No	None
7	Urothelial Carcinoma	Male	68.8	Recurrent	6	TURBT, gemcitabine + cisplatin	Pembrolizumab	PR	20.7	2	Left inguinal LN, left obduator LN	New	2500	5	Yes	SBRT x 2
8	HCC	Male	66.2	IV	4	None	Nivolumab	PR	9.1	2	Left crus, Left adrenal	Old	5000	5	Yes	SBRT
9	HCC	Female	64.9	IV	6	None	Nivolumab	PR	15	1	Liver	New	5000	5	No	None
10	HCC	Female	47.8	Recurrent	1	Surgery, TACE	Nivolumab	SD	7.7	1	Porta hepatis LN	New	4500	5	Yes	Lenvantinib, ramucirumab, cabozantinib

11	HCC	Male	59.4	Recurrent	2	Surgery , Y90	Nivolumab	CR	19.6	1	Right lung	New	5000	5	No	None
12	HCC	Female	64.7	Recurrent	3	TACE	Nivolumab	PR	4.5	1	Liver	New	4000	5	No	None
13	HCC	Male	33.6	Recurrent	3	Sorafenib, TACE, SBRT	Nivolumab	SD	10.9	1	Portal vein	New	5000	5	No	None
14	Urothelial Carcinoma	Male	49.0	IV	> 10	None	Pembrolizumab	SD	15.9	1	Celiac LN	New	5000	10	Yes	SBRT x 2, enfortumab vedotin
15	Urothelial Carcinoma	Male	71.7	Recurrent	3	Surgery, intravesical BCG, gemcitabine + carboplatin	Atezolizumab	PR	32.4	1	Left nephrectomy bed	Old	5000	5	Yes	Pembrolizumab + enfortumab vedotin
16	HCC	Female	61.7	Recurrent	5	Surgery	Nivolumab	SD	12.8	1	Extrahepatic mass	Old	5000	20	Yes	Y90, TACE, RFA
17	Adenocarcinoma	Male	59.0	Recurrent	4	Surgery, gemcitabine + cisplatin, FOLFIRINOX, Y90	Pembrolizumab	CR	14.9	1	Right adrenal gland	Old	4800	4	No	None
18	Adenocarcinoma	Female	60.8	Recurrent	3	ChemoRT with 5-FU, surgery	Pembrolizumab	PR	11.3	1	Para-aortic LNs	New	5500	25	Yes	SBRT
19	HCC	Male	59.4	IV	8	None	Pembrolizumab	CR	9.1	1	Liver	Old	5000	5	No	None
20	HCC	Female	64.7	Recurrent	1	TACE, surgery	Pembrolizumab	PR	32.8	1	Liver	Old	4500	15	No	None

21	HCC	Female	68.3	Recurrent	2	Surgery	Nivolumab	PR	20.8	1	Liver	Old	4500	5	No	None
22	Urothelial Carcinoma	Male	69.0	Recurrent	7	TURBT + intravesicular BCG, surgery	Nivolumab	CR	9.3	1	Left inguinal LN	New	3000	5	Yes	Pembrolizumab, SBRT
23	Colorectal Cancer	Male	40.3	Recurrent	> 10	Surgery	Nivolumab	CR	7.4	1	Lung	New	4800	3	No	None
24	Urothelial Carcinoma	Male	68.7	Recurrent	1	TURBT, gemcitabine + cisplatin	Pembrolizumab	SD	31.3	2	Left inguinal LN, left obduator LN	New	2500	5	Yes	SBRT
25	HCC	Male	58.9	IV	2	RT	Nivolumab	PR	3.5	2	Liver	New	4000	5	Yes	Lenvantinib
26	RCC	Female	55.1	Recurrent	4	Surgery, pazopanib, RT	Nivolumab	SD	5.4	1	Right kidney	Old	4800	3	Yes	Cabozantinib, Axitinib, Avelumab, Tivozanib
27	Melanoma	Male	54.3	IV	10	RT	Ipilimumab + Nivolumab	SD	5.3	1	Right intraparotid LN	Old	3000	5	No	None
28	Urothelial Carcinoma	Male	78.9	IV	> 10	Gemcitabine + cisplatin	Pembrolizumab	SD	16.4	2	Bladder and kidney	Old	6000	30	Yes	Enfortumab vedotin
29	NSCLC	Female	70.8	IV	2	RT, carboplatin + pemetrexed	Atezolizumab	SD	11.4	1	L3	New	1800	1	Yes	RT, pembrolizumab
30	HCC	Male	68.6	Recurrent	6	Y90, RT, sorafenib	Nivolumab	PR	15.7	1	Liver	Old	4500	5	Yes	RFA
